# Insights into the Role of Matrix Metalloproteinases in Precancerous Conditions and in Colorectal Cancer

**DOI:** 10.3390/cancers13246226

**Published:** 2021-12-10

**Authors:** Zahra Pezeshkian, Stefania Nobili, Noshad Peyravian, Bahador Shojaee, Haniye Nazari, Hiva Soleimani, Hamid Asadzadeh-Aghdaei, Maziar Ashrafian Bonab, Ehsan Nazemalhosseini-Mojarad, Enrico Mini

**Affiliations:** 1 Basic and Molecular Epidemiology of Gastrointestinal Disorders Research Center, Research Institute for Gastroenterology and Liver Diseases, Shahid Beheshti University of Medical Sciences, Tehran 19835-178, Iran; zahrapezeshkian@yahoo.com (Z.P.); peyravian.n@iums.ac.ir (N.P.); bahadorshojaee@ufl.edu (B.S.); hamid.asadzadeh@sbmu.ac.ir (H.A.-A.); 2 Department of Neurosciences, Imaging and Clinical Sciences, “G. D’Annunzio” University of Chieti-Pescara, 66100 Chieti, Italy; stefania.nobili@unich.it; 3 Center for Advanced Studies and Technology (CAST), University “G. D’Annunzio” Chieti-Pescara, 66100 Chieti, Italy; 4 Department of Microbiology, Faculty of Advanced Science and Technology, Tehran Medical Science, Islamic Azad University, Tehran 19395-1495, Iran; hani7311926@gmail.com; 5 Department of General Biology, Faculty of Fundamental Science, Islamic Azad University of Shahr-E-Qods, Tehran 37515-374, Iran; M_hivi@yahoo.com; 6 School of Medicine, University of Sunderland, City Campus, Chester Road, Sunderland SR1 3SD, UK; maziar.bonab@sunderland.ac.uk; 7 Gastroenterology and Liver Diseases Research Center, Research Institute for Gastroenterology and Liver Diseases, Shahid Beheshti University of Medical Sciences, Tehran 19835-178, Iran; 8 Department of Health Sciences, University of Florence, 50139 Florence, Italy; 9 DENOTHE Excellence Center, University of Florence, 50139 Florence, Italy

**Keywords:** Matrix Metalloproteinases (MMPs), polyp, colorectal cancer, TIMPs, MMP polymorphisms, MMP targeting

## Abstract

**Simple Summary:**

Colorectal cancer (CRC) is one of the most common cancer worldwide. CRC is derived from polyps and many factors, such as Matrix Metalloproteinases (MMPs) can gain the progression of colorectal carcinogenesis. Many investigations have indicated the role of MMPs in CRC development while there is not enough knowledge about the function of MMPs in precancerous conditions. This review summarizes the current information about the role of MMPs in polyps and CRC progression.

**Abstract:**

Colorectal cancer (CRC) is the third and second cancer for incidence and mortality worldwide, respectively, and is becoming prevalent in developing countries. Most CRCs derive from polyps, especially adenomatous polyps, which can gradually transform into CRC. The family of Matrix Metalloproteinases (MMPs) plays a critical role in the initiation and progression of CRC. Prominent MMPs, including MMP-1, MMP-2, MMP-7, MMP-8, MMP-9, MMP-12, MMP-13, MMP-14, and MMP-21, have been detected in CRC patients, and the expression of most of them correlates with a poor prognosis. Moreover, many studies have explored the inhibition of MMPs and targeted therapy for CRC, but there is not enough information about the role of MMPs in polyp malignancy. In this review, we discuss the role of MMPs in colorectal cancer and its pathogenesis

## 1. Introduction

At approximately 11% of all diagnosed cancer cases, CRC is the third most common cancer and the second most lethal cancer worldwide [1,2]. It is today well known that several factors contribute to the CRC pathogenesis, driving complex genetic and epigenetic processes that, ultimately, transform normal colonic mucosa to cancerous tissue [3]. CRC may initiate from benign polyps with the mucosal origin and can develop into carcinoma. Colorectal polyps, especially adenomas, are proliferative lesions that have been defined as the precursor of CRC. Therefore, the early detection and removal of these polyps can interrupt the progression of the adenoma-carcinoma sequence [4,5].

Many molecular signaling pathways are involved in CRC initiation and progression, such as ERK/MAPK, TGF-β, PI3K/Akt, Src/FAK, and β-catenin pathways. These pathways can promote the hallmarks of cancer such as inflammation, angiogenesis, metastasis, and invasion, also via the activation and overexpression of MMPs [6,7]. Thus, MMPs have been suggested as potential prognostic factors for the malignancy risk of colorectal polyps. MMPs are proteolytic enzymes implicated in the degradation of stromal connective tissues and of the extracellular matrix (ECM), a complex network that plays a key role in sustaining signaling transduction and thus cancer development and progression [8]. As such, MMPs have key roles in tumor initiation, progression, and metastasis and can affect tumor cell behavior by cleaving proapoptotic agents and producing an aggressive phenotype [9]. Because of these roles, MMPs have been detected as biomarkers in CRC progression [10]. A new challenge in CRC treatment is finding an effective pharmacological and therapeutic method for suppression of MMPs and targeted therapy of CRC [11]. This review will deal with the role of MMPs in colorectal carcinogenesis from colorectal polyps to CRC.

## 2. CRC Pathogenesis and Molecular Classification

Colorectal polyps result from atypical cell proliferation in the colorectal tissue. Based on histological and morphological features, colorectal polyps are divided into neoplastic (adenoma) and non-neoplastic (hyperplastic, hamartomatous, and inflammatory) types [5,12]. Neoplastic polyps, also known as adenomatous polyps, are subclassified by their histological characteristics as tubular, villous, or tubulovillous adenomas. Previous investigations demonstrated that approximately 5–10% of neoplastic polyps are villous adenomas and most of them show dysplasia. Approximately 10–15% of neoplastic polyps show morphological features of both villous and tubular types [13]. Adenomas are not usually transformed to carcinoma, but there is evidence that the adenoma-carcinoma sequence originates from adenomatous polyps [14]. Also, hyperplastic polyps may possess malignancy potential [15]. CRC is caused by the misregulation of some oncogenes such as *KRAS* and *c-MYC* and tumor suppressor genes such as *P53* and APC, which control cellular signal transduction [16,17,18].

### 2.1. Molecular Mechanism of CRC

Specific features characterize CRC and its pathogenesis based on genetic, epigenetic, and transcriptomic factors. Three main molecular abnormalities are involved in CRC carcinogenesis:

- Microsatellite instability (MSI): it consists of mutations in DNA mismatch repair (MMR) genes such as *MSH2*, *MLH1*, *PMS2*, *MLH3*, *MSH3*, *PMSI*, and *EXO1*; MSI is rare in polyps but it is always found in serrated polyps and about 15–20% of all CRC cases are derived from MSI [19,20].
- Chromosomal instability (CIN): this abnormality is identified in 85% of CRC cases and consists of a gain (1q, 7p, 8q, 13q, 2pq) or loss (8q, 15q, 17p, 18p) of chromosomal genes, activation of proto-oncogenes (*KRAS*, *SRC*, *c-MYC*), and inactivation of tumor suppressor genes (*P53*, *APC*) [21].
- CpG Islands Methylator Phenotype (CIMP): these regions, located in the gene promoter, could disturb the activation of tumor suppressor genes. CIMP phenotype is represented by hypermethylation of CpG dinucleotides and premalignant serrated polyps are correlated with CIMP [22,23]

### 2.2. Molecular Classification Based on Transcriptomic Analysis

Based on gene expression profiles, CRC has been classified into subgroups with distinct molecular and clinical features [24].

- Consensus molecular subtype (CMS) classification: CMS classification provides biological insight into metastatic colorectal cancer (mCRC) carcinogenesis and predicts CRC prognosis [25].
  ◦ CMS1 (14%) indicates MSI, CIMP, and *BRAF* mutation and immune activation.
  ◦ CMS2 (37%) shows Wingless-Type MMTR integration site family member (WNT), MYC signaling activation, and epithelial involvement.
  ◦ CMS3 (13%) demonstrates MSI, CIMP, and *KRAS* mutations and metabolic involvement.
  ◦ CMS4 (23%) includes invasion, metastatic situations, and TGF-β signaling co-activation and angiogenesis. Also, epithelial-mesenchymal transition (EMT) is a crucial event in colorectal carcinogenesis and is involved in CMS4 status. EMT can result in advanced-stage CRC, poor patient survival, and worst clinical features [26,27] and CMS4 subgroup shows the most unfavorable prognosis.
- CRC intrinsic subtypes (CRIS): CRIS is a unique classification exclusively based on the cancer cell-specific transcriptome of CRC since the extrinsic factors of the stroma have not been analyzed. It classifies CRC into five novel transcriptional groups that, thus, further clarify biological understanding of CRC heterogeneity.
  ◦ CRIS-A is enriched for *BRAF*-mutated MSI tumors and *KRAS*-mutated MSS tumors that are without targeted therapeutic options.
  ◦ CRIS-B is related to invasive tumors with poor prognosis and high TGF-ß signaling. CRIS-B is unconnected to the CMS4 mesenchymal subtype, which also indicates aggressive tumors with TGF-ß pathway activation.
  ◦ CRIS-C is dependent on EGFR signals and is sensitive to anti-EGFR monoclonal antibody treatment.
  ◦ CRIS-D shows IGF2 overexpression. This occurrence has been involved in desensitization to the EGFR blockade in patients with *KRAS* wild-type tumors.
  ◦ CRIS-E indicates *KRAS*-mutated, Paneth cell-like CIN tumors refractory to anti-EGFR antibody treatment [28].

## 3. Structure and Function of MMPs

MMPs are a family of zinc-dependent endopeptidases consisting of a propeptide sequence, a catalytic domain, a hinge region, and a hemopexin (PEX) domain [29]. The propeptide domain is highly conserved and can regulate the sequence that interacts with Zn^2+^. Also, cystine within this area permits the MMPs to be in the active or inactive status [30]. The catalytic domain possesses a conserved zinc-binding motif which, in the active condition, will disconnect from the propeptide domain. Movement between the catalytic and PEX domain is done via hinge regions [29]. According to their structural domains, MMPs have been categorized into collagenase, gelatinase, stromelysin, matrilysin, and membrane-bound MMPs (MT-MMPs) [31,32].

**Figure 1:**
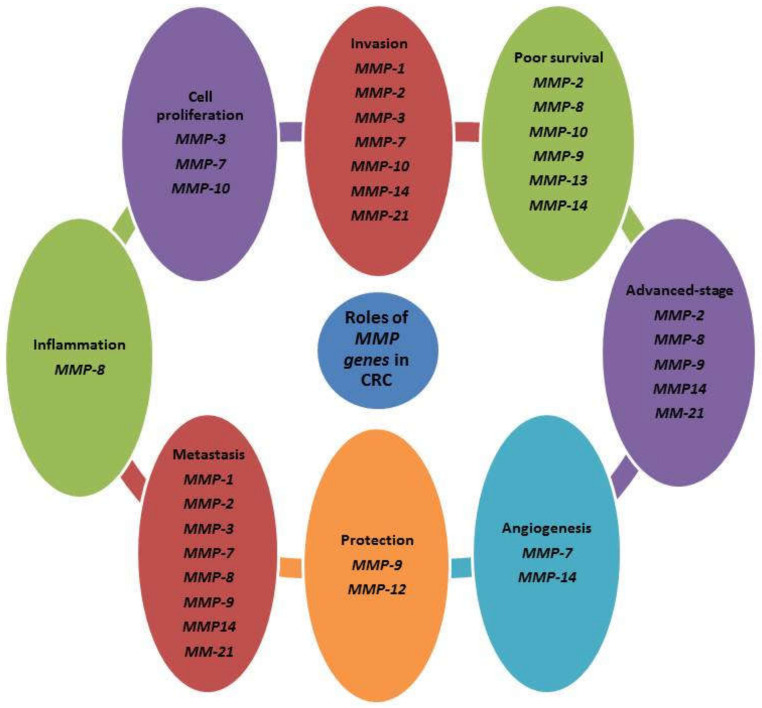
Summary of the prominent *MMP* genes in CRC. MMPs play different functions in CRC.

**Figure 2:**
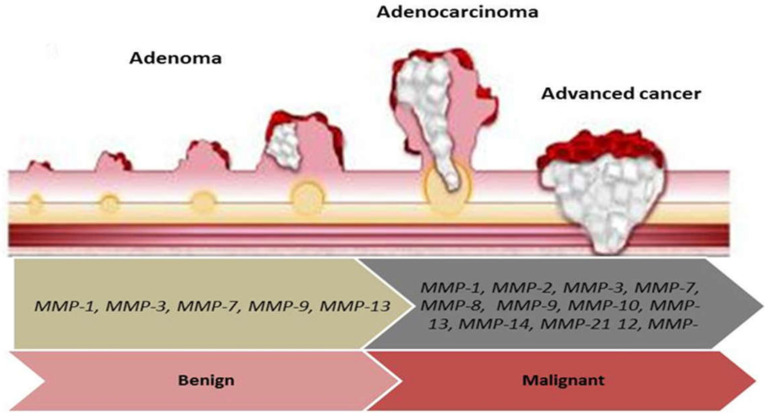
The diagram indicates the role of *MMPs* genes in adenoma development, colorectal adenoma-carcinoma sequence, and tumor progression. *MMP-1*, *MMP-3*, *MMP-7*, *MMP-9*, and *MMP-13* are involved in adenoma development. *MMP-1*, *MMP-2*, *MMP-3*, *MMP-7*, *MMP-8*, *MMP-9*, *MMP-12*, *MMP-13*, *MMP-14*, and *MMP-21* participate in adenoma-carcinoma sequence and tumor progression.

MMPs play a crucial role in the remodeling of the ECM by digestion of ECM components, stimulation of cell surface proteins. Also, they can control the activity of other proteinases, growth factors, chemokines, and cell receptors, and moderate many biological functions [33]. MMPs can regulate cellular growth, migration, survival, and adhesion in biological and pathological statuses (Table 1, Figure 1). Due to the MMP’s key roles, the dysregulation of their expression levels and their activation lead cancerous cells to proliferation, angiogenesis, survival, invasion, malignant transitions, and immune dysregulation [34,35,36]. Also, the tissue inhibitors of metalloproteinase (TIMPs) control the activation of MMPs and have a critical action in precancerous conditions, CRC progression, and metastasis (Table 2, Figure 2) [11,37].

## 4. The Function of MMPs in Colorectal Polyps and Cancer

### 4.1. MMP-1, MMP-13, and MMP-8 (Collagenases)

The specific targets for MMP-1 and MMP-13 are in the intestine. MMP-1 can digest type I, II, III, VII, VIII, X collagen, and gelatin. Upregulation of *MMP-1* gene was detected in CRC patients compared to normal tissue [6,59]. Eiro et al., found overexpression of *MMP-1* gene in serrated, villous, and tubulovillous adenomas (i.e., polyps with high potential for transformation to CRC) [44]. Previous investigations demonstrated the correlation between *MMP-1* gene expression and CRC progression: high expression levels of *MMP-1* were associated with invasion, advanced stage metastasis, LNM, and shorter overall survival [60,61]. Wang, et al. investigated the role of MMP-1 in the development of CRC. They found that the downregulation of MMP-1 expression inhibited the progression of CRC in vitro and in vivo by suppressing the PI3K/Akt/c-myc signaling pathway and the EMT [6].

MMP-13, another member of the collagenase category, could degenerate type III collagen. According to the strength of the association between pathologic stage and immunoreactivity scoring (IRS) of MMP-13, in high-grade adenomas and CRC, MMP-13 was observed with a moderate and strong staining intensity, respectively [46]. This result indicated that MMP-13 could help to predict metastatic behavior and prognosis of early-stage cancerous and precancerous colorectal adenoma [46,62]. The study of the association between grade dysplasia and MMP-13 expression in 137 biopsies from patients with cancerous and non-cancerous colorectal adenomas showed that the high expression level of MMP-13 IRS could be helpful to predict metastatic state, prognosis, and recrudescence at an early stage of cancerous and precancerous colorectal adenoma. Moreover, the upregulation of MMP-13 IRS from low to high-grade adenoma was considered an early predictive cancer biomarker [46]. Meanwhile, several studies confirmed that upregulation of MMP-13 was related to advanced CRC and liver metastasis [62,63,64]. Also, the expression of MMP-13 on the primary tumor cell surface is increased in inflammatory bowel disease. The expression of MMP-13 is closely related to the progression, early relapse, and high mortality of CRC [63,65].

Another member of collagenase enzymes is MMP-8 which is frequently expressed by neutrophils. MMP-8 cleaves many substrates, such as type I, II, and III collagen. This MMP is mainly considered to play a protective role against cancer. However, more recent findings also suggest an oncogenic function of *MMP-8* gene [66,67].

Sirnio et al., found that enhanced-serum MMP-8 level in CRC patients was significantly related to advanced-stage CRC, distant metastasis, lack of MMR, and poor survival. Thus,, they evidenced that MMP-8 is correlated with inflammation and CRC progression [68].

### 4.2. MMP-2 and MMP-9 (Gelatinase)

MMP-2 and MMP-9, known as gelatinases, can digest type IV collagen and gelatin [69]. Murname et al. showed that MMP-2 protein activity in adenomas with high-grade dysplasia (HGD) was different from adenomas with low-grade dysplasia (LGD). They suggested that the active *MMP-2* gene could predict CRC malignancy risk in patients with adenomatous polyps [70]. Some studies also indicated high expression levels of MMP-9 protein in adenomas with HGD compared to adenomas with LGD and normal tissue. As such, researchers speculated that upregulation of MMP-9 is a primary event in the CRC adenoma-carcinoma sequence [41,71]. High expression levels of MMP-2 protein in CRC tumors compared to normal mucosa have also been reported [41,72]. In addition, a statistically significant relationship between upregulation of *MMP-2* gene with advanced-stage CRC or CRC progression has been observed [41,73,74,75]. On this basis, *MMP-2* has been suggested as a potential biomarker to detect CRC progression and predict patient survival. Furthermore, overexpression of the *MMP-2* gene was associated with metastasis of lymph nodes and a decrease of cell adhesion in tumors [73].

Finally, also the upregulation of *MMP-9* gene was associated with the advanced stage of CRC and suggested as a biomarker predictive of poor overall survival [41,76]. Chen et al. indicated that the overexpression of *MMP-9* gene promoted CRC metastasis through the MKK-3/p38/NF-κB pro-oncogenic pathway. Furthermore, they suggested *MMP-9* gene as a potential molecular target for targeted therapy to treat metastatic CRC patients [76].

On the contrary, some investigations reported that *MMP-9* gene has a protective role in CRC by stimulating Notch activation resulting in the activation of p21WAF1/Cip1 leading to the suppression of β-catenin [77,78]. In a recent study, although in colitis-associated colon cancer, Walter et al. confirmed this observation by revealing that MMP-9 protein expression was associated with reduced ROS levels, decreased DNA damage, and stimulated mismatch repair pathway [79].

In an interesting study, Wei et al., by analyzing microbiota in tumors obtained by patients with different prognoses, found that the expression of some inflammatory genes, including *MMP-9*, was associated with the abundance of specific bacteria. High levels of *MMP-9* expression were significantly correlated with the high abundance of *B. fragilis* and *F. nucleatum* whereas a high level of F*. prausnitzii* was associated with downregulation of *MMP-9* [80].

### 4.3. MMP-3, MMP-10 (Stromelysin)

Another member of MMPs family is MMP-3, or stromelysin-1, which degrades collagen (types II, III, IV, IX, and X), proteoglycans, fibronectin, laminin, and elastin in ECM.

Sipos et al., found a positive association between MMP-3 protein expression and the adenoma–dysplasia–carcinoma sequence. In particular, they reported that high-grade dysplastic sessile adenomatous-stage and early-stage CRC conditions can be differentiated based on the stroma expression of MMP3 [81]. Meaningful positive associations between the protein expression level of MMP-3, invasion, lymph node metastasis, histological type of CRC, and poorly differentiated tumor were reported by Islekel et al. [82]. MMP-3 can activate other MMPs, such as MMP-1, MMP-7, and MMP-9, to promote the progression of tumor initiation [83,84].

MMP-10 also belongs to the stromelysin family. It can digest collagen types II, III, IV, IX, X, proteoglycans, fibronectin, laminin, and elastin. Also, MMP-10 enhances cell growth and invasion in CRC, and its upregulation was found to be associated with poor survival [49,85].

### 4.4. MMP-7 (Matrilysin)

MMP-7, or matrilysin, digests fibronectin, laminin, type I collagen, and gelatin. It can provide the right condition for vascularization via cleavage of ECM [86]. A major ratio of MMP-7 expression in tumor cells has been reported. Qasim et al., found MMP-7 protein overexpression in villous adenomatous polyps compared to other types of polyps and demonstrated that MMP-7 protein overexpression is an initial event in CRC carcinogenesis that could lead adenomas to CRC [53]. In our laboratory, we observed high expression levels of *MMP-7* and *VEGF-A* mRNA in adenomatous polyps compared to normal tissue. We found that the expression levels of *MMP-7* and *VEGF-A* genes were higher in villous adenoma than in other types of adenomas. Thus, we concluded that the *MMP-7* gene overexpression has a critical role in colorectal adenoma angiogenesis and could be a primary event in the adenoma-carcinoma sequence [45].

*MMP-7* gene can enhance tumor growth and metastasis [87]. Also, MMP-7 activates other MMPs, such as proMMP9 and proMMP2 [88] In addition. MMP-7 exerts a wide spectrum of activities not only as an enzyme but also as a signaling molecule. In fact, it has been shown that MMP-7 trans-activates EGFR by releasing the heparin-binding epidermal growth factor (HB-EGF) in CRC cells, with consequent cell proliferation and apoptosis regulation [89,90].

### 4.5. MMP-12 (Metalloelastase)

MMP-12, or metalloelastase, can digest different substrates. Several studies considered *MMP-12* gene as an anti-metastatic agent [91,92]. Also, it could inhibit angiogenesis by downregulation of *VEGF* and enhancement of the endogenous angiogenesis inhibitor angiostatin. Overall, the role of *MMP-12* in tumor suppression and increase in overall survival has been widely recognized [93,94,95].

Importantly, Klupp et al., found higher levels of MMP-12 protein expression in sera of CRC patients compared with those of healthy individuals. Also, they suggested an association between MMP-12 protein expression levels and CRC advanced disease and vascular invasion. Furthermore, a significant correlation between the upregulation of MMP-12 expression and poor survival was shown [49].

### 4.6. MMP-21 (XMMP)

MMP-21 (XMMP) can degenerate aggrecan (cartilage-specific proteoglycan core protein) in the internal region of ECM [96]. Overexpression of MMP-21 protein in CRC compared with normal tissue was shown in many studies [97,98]. Furthermore, significant associations between MMP-21 protein expression and CRC tumor invasion, lymph node metastasis, and distant metastasis were found [97,99]. Wu et al., showed that MMP-21 not only affected CRC progression but also was an independent prognostic biomarker in patients with stage II and stage III CRC cancer. Taken together, these facts led them to conclude that MMP-21 could be used for targeted therapy in CRC [97]. Huang et al., demonstrated that the upregulation of MMP-21 protein was related to shorter overall survival in patients with CRC [98].

### 4.7. MMP-14 (MT1-MMP)

MMP-14, called MT1-MMP, acts on matrix substrates, such as collagens I, II, III, and gelatin. The *MMP-14* gene plays a crucial role in many biological and pathological conditions and activation of proMMP2 [92,100]. The role of *MMP-14* in angiogenesis and cancer invasion has been identified by previous investigations [101,102,103]. Cui et al., observed statistically significant associations between the overexpression of *MMP-14* gene in CRC compared to normal mucosa. Their analysis indicated that high expression levels of *MMP-14* were associated with advanced-stage CRC, lymph node metastasis, and poor overall survival. They concluded that the *MMP-14* gene is an oncogene and may represent a potential prognostic biomarker in CRC [104].

Yang et al., showed in an in vivo CRC model that the STAT3 phosphorylation activity and the overexpression of MMP14 protein were enhanced by the overexpression of Hes1 gene. Also, they suggested that Hes1 promoted the invasion of colorectal cancerous cells via the STAT3-MMP14 pathway [103]. It was reported that the overexpression of MMP-14 protein was associated with Prox1 gene. When Prox1 gene was deleted, MMP14 protein was increased, and the mice showed slow-growing, matrix-rich, chemotherapy-resistance, and cancerous cells with malignant stromal features, including activation of fibroblasts, blood vessels dysfunction, and lack of cytotoxic T cells [105].

## 5. The Effects of Polymorphisms of *MMP* Genes on Colorectal Carcinogenesis

Single-nucleotide polymorphisms (SNPs) are a common genetic variation involving a single base pair in DNA. SNPs are mostly located in the gene promoter region and may have an impact on gene and protein expression levels. The effects of MMP polymorphisms have been observed in many cancers such as CRC and hepatocellular carcinoma [106,107].

In a Japanese population, the *MMP-1* 1G/2G polymorphism was detected and associated with the development of CRC [108]. In the Iranian population, Kouhkan et al., demonstrated that *MMP-1* 2G/2G genotype polymorphism was correlated with invasion risk of CRC, especially in smoker men [109]. In the Netherlands, *MMP-2*-1306C>T SNP was detected in CRC patients, and the T/T genotype was found to be associated with poor overall survival whereas C/C and C/T genotypes showed better outcomes. No difference in overall survival was instead observed among patients with different genotypes of the *MMP-9*-1562C>T SNP [110]. Also, in a cohort study of Taiwanese CRC patients, Ting et al. indicated that patients carrying the A/A genotype of the *MMP-2*-1575G>A SNP had a higher risk to develop distant metastasis compared with patients carrying the T/T genotype [111]. In a Polish population with CRC, individuals with the G/G variant genotype of *MMP-7*-181A>G SNP had a higher risk of lymph node involvement and advanced tumor infiltration than patients carrying the A/A genotype [112]. A Chinese study showed that the *MMP-9* R279Q SNP relative to the R/R genotype was correlated with a higher risk of CRC compared with the QQ genotype. Also, the allele frequency of the *MMP-1* 16071G/2G and *MMP-7* 181 A/G polymorphisms were not associated with CRC [113]. In a Korean population, the homozygous *MMP-9*-1562C/C genotype was significantly more frequent in CRC cases than in the control group [114]. In Sweden, researchers found that the A/A genotype of *MMP-12*-82A>G increased the risk of disseminated malignancy in CRC patients while the A/A genotype of *MMP-13*-82A>G was not correlated to invasion [115].

Lièvre et al., investigated *MMP-3*, *MMP-7*, and *MMP-1* genes promoter polymorphisms in 295 patients with large adenomas and 302 patients with small adenomas. The analysis revealed a significant association between *MMP-3*-1612 ins/del A, *MMP-1*-1607 ins/del G polymorphism, and small adenomas; also, adenomas were associated with the combined genotype 2G/2G-6A/6A. However, no significant association between *MMP-7* polymorphism and the development of adenomas was found. The authors suggested that only the study *MMP-3* and *MMP-1* gene promoter polymorphisms had potential roles in the development of adenomas from normal colon epithelial cells or in the earliest steps of CRC [57].

Tai et al., showed that *MMP-8* rs11225395 related to the risk of CRC and worst outcomes in a subpopulation of the Han Chinese population. On this basis, they suggested *MMP-8* rs11225395 polymorphism as a potential biomarker predictive of CRC susceptibility [116].

## 6. Targeting MMPs in CRC Treatment

### 6.1. Pharmacological Inhibition

Several pharmacological inhibitors of MMPs (MMPIs) have been studied and tested in phase I-III clinical trials, but to date, none of these drugs has been approved for the treatment of cancer, including CRC. Overall, the late stages of the clinical experimentation failed because of the substantial toxicity and weak selectivity of MMPIs [117]. Mainly, candidate MMPIs are represented by small molecules, peptides, and antibodies [118]. Currently, only one broad-spectrum MMPI has been approved by FDA but it has not indication in cancer (i.e., the small molecule periostat) [117,119]. Other MMPIs, such as the small molecule prinomastat, selective for MMP-1, MMP-2, and MMP-9 [120,121,122,123] and the GA-5745/andecaliximab, a selective anti-body against MMP-9, have reached the phase III [124,125]. However, none of these trials includes CRC.

### 6.2. Inhibition of MMPs by TIMPs

**Figure 3:**
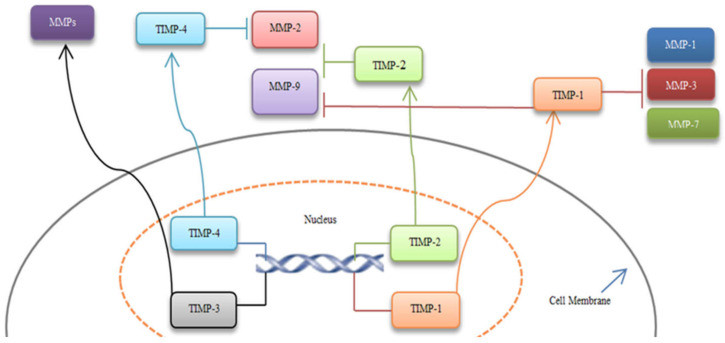
MMPs inhibition by TIMPs. TIMP-1 inhibits MMP-1, 3, 7, 9. TIMP-2 can suppress MMP-2 and 9, and TIMP-4 blocks MMP-2. These inhibitions result in the primary tumor transitioning to advanced CRC. Moreover, TIMP-3 has a protective effect on CRC cases and could bind to several MMPs [126,127,128].

Since MMPs are naturally inhibited by TIMPs, these proteins have also been widely investigated mainly to exploit their ability to discover potential strategies for MMP inhibition [126]. The TIMP family consists of four members of proteins (TIMP1-4) that form a 1:1 complex with MMPs. Dysregulation of this complex due to the increased expression of MMPs or a decreased control by TIMPs has been observed in several diseases, including cancer. TIMPs control the activity of MMPs via binding to them (Figure 3) [126,127,128].

TIMP-1 inhibits MMP-1, 3, 7, 9 and affects angiogenesis [37,129]. Previous investigations considered a dual activity for the *TIMP-1* gene: in particular, *TIMP-1* was associated with tumor growth at the early stages of colon cancer, and decreased activity of *TIMP-1* could lead to tumor invasion [130,131].

TIMP-2 can suppress MMP-2, MMP-9, and microvascularization [129,132]. Also, downregulation of TIMP-2 is related to invasive CRC [133]. Wang et al., reported that downregulation of *TIMP-2* in CRC tumor tissues was meaningfully correlated with the depth of invasion, lymph node metastasis, tumor stage, and poor survival [134].

*TIMP-3* is known as a tumor suppressor gene and inhibits several MMPs. *TIMP-3* downregulation is associated with advanced CRC [135]. Lin et al., represented that, adenovirus-mediated *TIMP-3* transduction in CT26 colon cancer cell line suppressed cell growth and stimulated apoptosis. Also, *TIMP-3* transduction inhibited migration and invasion. In vivo data indicated that *TIMP-3* prevented in vivo tumor growth and liver metastasis [136].

TIMP-4 protein suppresses MMP-2, and one study showed that overexpression of TIMP-4 increased the survival rate of rectal cancer [128].

Currently, no drug mimicking the TIMP activity has been obtained as well as no gene therapeutic approach able to modulate the activity of TIMPs is available.

### 6.3. MMPs Regulation by microRNA

MicroRNAs, a class of small, endogenous RNAs of 21–25 nucleotides in length, control gene and protein regulation via binding and digesting target mRNA (Table 3). Suppression of MMPs by microRNAs is a suggested way for CRC treatment. Some evidence has been provided. In particular, microRNA-34 (miR-34a) plays a role as a tumor suppressor, and its overexpression could suppress *MMP-1*, *MMP-9*, and tumor cell proliferation, migration, and invasion via acetylation of *P53* in CRC [137,138,139]. The upregulation of miR-139 reduces proliferation, migration, and invasion by suppression of the IGF-IR/MEK/ERK signaling and *MMP-2* gene in CRC patients [140]. Upregulation of miR-29a increases CRC metastasis via suppression of KLF4 (Kruppel-like factor 4), transcription factor, and upregulation of *MMP-2* gene [141]. Also, miR-29b suppresses CRC metastasis, reduces angiogenesis and EMT by targeting the *MMP-2* gene [142]. Overexpression of miR-143 can suppress the *MMP-7* gene directly and prevent colorectal tumor cell proliferation and invasion [143].

### 6.4. MMPs Regulation by Long Non-Coding RNAs

Long non-coding RNAs (lncRNAs) can regulate gene expression and have key roles in cell proliferation, migration, invasion, apoptosis, metastasis, and EMT in CRC. In this regard, lncRNA-targeted therapy is today considered a potential promising strategy for CRC treatment [144]. In fact, based on mechanistic studies investigating the complex lncRNA-mediated sponge interactions in CRC, potential therapeutic targets for the treatment of this cancer may be identified. Among the available findings, Tian et al., demonstrated that the suppression of *TUG1* by shRNA prevented *MMP-14* expression, proliferation, invasion, and EMT in colon cancer [145]. Sun et al., found a significant association between XIST inhibition and suppression of *c-Myc*, *cyclinD1,* and *MMP-7* expression through inactivation of Wnt/β-catenin signaling pathway [146]. A recent investigation showed a meaningful correlation between the overexpression of LINC00963 and the upregulation of *MMP-2* and *MMP-9*, proliferation, migration, and invasion of CRC cells [147]. Duan et al., revealed that the inhibition of the CCEPR lncRNA reduced the expression levels of *MMP-2* and *MMP-9*, and prevented EMT in CRC cells [148]. Pan et al., realized that the expression level of MMP-2 protein was notably decreased when PCA3 was knocked out. In addition, suppression of PCA3 inhibited colon cancer cell invasion and migration [149].

## 7. Conclusions

In summary, MMPs genes and proteins, through complex mechanisms involving the induction of many molecular signaling pathways and the EMT process, play a relevant role in the transition from pre-cancerous lesions and polyps to advanced CRC. However, further investigation is needed to understand how MMPs exactly work. This would improve the selectivity of MMPIs that could be exploited in a dual-mode: to treat CRC alone or in combination with targeted agents and/or chemotherapy and to prevent CRC development.

## Figures and Tables

**Table 1:** Matrix Metallopeptidases Features in Humans.

MMP Gene	Chromosomal Location	Enzyme	Substrate
*MMP-1*	11q22.2	Collagenase-1	Col I, II, III, VII, VIII, X, Gelatin
*MMP-8*	11q22.2	Collagenase-2	Col I, II, III, VII, VIII, X, Gelatin, Aggrecan
*MMP-13*	11q22.2	Collagenase-3	Col I, II, III, VII, VIII, X, Gelatin
*MMP-2*	16q12.2	Gelatinase A	Gelatin, Col I, II, III, IV, VII
*MMP-9*	20q13.12	Gelatinase B	Gelatin, Col IV, V
*MMP-3*	11q22.3	Stromelysin-1	Col II, III, IV, IX, X, proteoglycans, fibronectin, laminin, and elastin.
*MMP-10*	11q22.2	Stromelysin-2	Col II, III, IV, IX, X, proteoglycans, fibronectin, laminin, and elastin
*MMP-7*	11q22.2	Marilysin-1	Fibronectin, Laminin, Col I, Gelatin
*MMP-14*	14q11.2	MT-MMP	Gelatin, Fibronectin, Laminin
*MMP-12*	11q22.2	Metalloelastase	Gelatin, Fibronectin, Col IV
*MMP-21*	10q26.2	XMMP	Aggrecan

**Table 2:** Summary of Investigations about the Roles of MMP Genes and Proteins in Colorectal Polyps and Cancer.

References	Gene/Protein Expression	Samples	Methods	Results
Huang X., et al., 2021 [38]	MMP-7, MMP-9, MMP-11, TIMP-1, TIMP-*2, CEA*	Human polyps and tumor	Enzyme-linked immunosorbent assay	A combined detection model, including MMP-7, TIMP-1, and CEA improved both the specificity and sensitivity for detecting CRC.
Zhou X., et al., 2021 [39]	MMP-7, MMP-9, MMP-11, TIMP-1, TIMP-*2, CEA*	Human CRC	ELISA and electrochemiluminescence immunoassay	The miR 135a was downregulated and MMP 13 was increased in samples. Combined detection of the two had a good diagnostic effect on the occurrence of CRC.
Rasool M., et al., 2021 [40]	TGF, VEGF, TNF, ILs, MMP-2, 9, 11, and 19	Human polyps and tumor	ELISA	Significant upregulation of MMP-2, MMP-9, MMP-11, and MMP-19 was reported in polyp and colon cancer samples compared with their MMP profile in normal samples.
Barabás L., et al., 2020 [41]	MMP-2, MMP-7, MMP-9, TIMP-1 and TIMP-2	Human adenomas, and CRC	ELISA	The serum antigen concentrations of MMP-7, MMP-9, TIMP-1, and TIMP-2 were significantly increased in patients with CRC and adenomas compared with the controls. They were also activated in premalignant adenomas.
Hsieh S.L., et al., 2019 [42]	Study of the mechanism of carnosine, TIMP-1, and *MMP-9*	Human HCT-116 CRC cell line	MTT assay and qPCR	The carnosine inhibits the migration and intravasation of human CRC cells. The regulatory mechanism may occur by suppressing NF-κB activity and modulating MMPs and EMT-related gene expression in HCT-116 cells treated with carnosine. *MMP-9* mRNA and protein levels were decreased. *TIMP-1* mRNA and protein levels were increased.
Kıyak R., et al., 2018 [43]	MMP-7, COX-2, TIMP-1, and CEA protein	Human polyps	ELISA and chemiluminescent enzyme immunometric assay (CEIA)	The plasma TIMP-1 levels were significantly elevated in cancer compared with the polyp group. The plasma MMP-7 levels were decreased in polyps compared with the control group. The plasma CEA and TIMP-1 are valuable biomarker candidates for differentiating CRC from colorectal polyps.
Eiró N., et al., 2017 [44]	*MMP-1*, *2*, *7*, *9*, *11*, *13* and *14*	Human adenomas and hyperplastic polyps	Real-time PCR and Western-blot, and	The hyperplastic polyps had the lowest levels of *MMP-1* and *MMP-7*. Tubular polyps had high levels of both *MMP-7* and *MMP-14*, and tubulo-villous adenomas had high levels of *MMP-1, 7*, and *14* compared with the normal group.
Pezeshkian Z., et al., 2017 [45]	*MMP-7* and *VEGF-A*	Human adenomas	Real-time PCR in 50 biopsy samples of adenomas including villous, tubular, and tubulo-villous types, and 20 paired tissue samples	The *MMP-7* mRNA expression was significantly higher in villous adenoma with high-grade dysplasia compared with the control group. *MMP-7* and *VEGF-*A are prognostic biomarkers for colorectal adenoma polyp progression to malignancy.
Wernicke A.K., et al., 2016 [46]	Association between grade of dysplasia and MMP-13 expression	Human adenomas and hyperplastic polyps	Immunohistochemistry and immune-reactive score (IRS)	The MMP-13 has been identified as an excellent marker of high-grade intraepithelial neoplasia and CRC. The strength of the association between pathologic stage and immune-reactive MMP-13 scoring emphasizes its potential for diagnosis in precancerous colorectal lesions.
Gimeno-García A., et al., 2016 [47]	MMP*-9*	Patients’ blood, adenomas, hyperplastic polyps, and CRC tissue	Luminex XMAP technology, gelatin zymography, western blot, and SNP analysis in 150 blood and tissue	There was a significant correlation between plasma and tissue levels of MMP-9. Plasma MMP-9 levels in patients with neoplastic lesions were significantly higher than in healthy controls. Also, MMP-9 in CRC was higher than in non-advanced adenomas.
Annaha’zi A., et al., 2016 [48]	MMP-9	Patients′ stool samples, adenomas, hyperplastic polyps, and CRC tissue	ELISA	Stool MMP-9 was significantly increased in CRC compared with all the other groups. Stool MMP-9 may be a new noninvasive marker in CRC.
Klupp et al., 2016 [49]	MMP-7, MMP-10, and MMP-12	Serum specimens of patients with colon adenocarcinoma	Luminex based multiplex assay	Expression levels of MMP-7, MMP-10, and MMP-12 in serum of colon cancer patients are different compared with serum specimens of the healthy control group. The upregulation of MMP-7, MMP-10, and MMP-12 in colon cancer patients’ serum was associated with a poor prognosis.
Otero-Estévez O., et al., 2015 [50]	MMP-9	Human adenomas and CRC	non-invasive stool immunochemical test (FIT) and ELISA	The MMP-9 levels were higher in advanced adenomas and CRC compared with those reported in samples of healthy individual. Elevated MMP-9 concentration was associated with several lesions, size, and adenoma histology.
Bengi G., et al., 2015 [51]	*MMP-7*, *TIMP-1*, and *COX-2*	Human adenomas and CRC	Real-time PCR	The expression of *TIMP-1*, *COX-2*, and *MMP-7* was significantly higher in polyps compared with normal tissue. Overexpression of MMP-7, COX-2, and TIMP-1 determine an important role of these genes in the progression of colon cancer.
Odabasi M., et al., 2014 [52]	MMP-9 and NGAL	Human adenomas and CRC	Immunohistochemistry	The MMP-9 and NGAL overexpression in neoplastic polyps might be used as markers to separate them from non-neoplastic polyps. These genes as immune-histochemical markers determine dysplasia in the early steps of the colorectal adenoma-carcinoma sequence.
Qasim B.J., et al., 2013 [53]	MMP-7	Human adenomas	Immunohistochemistry	MMP-7 was expressed in advanced colorectal adenomatous polyps with large size, severe dysplasia, and villous.
Sheth R.A., et al., 2012 [54]	MMP-2, and MMP-9	Xenograft model of CRC in nude mice	The MMP enzyme activity was measured by an enzyme-activatable optical molecular probe and quantitative fluorescence colonoscopy in nude mice which received celecoxib versus vehicle	There was an apparent linear relationship between measured MMP activity and tumor growth rate.
Murname M.J., et al., 2009 [55]	MMP-2 and MMP-9	Mouse models of CRC and human HT-29 CRC cell line	Gene-expression microarray and ELISA	The plotted receiver operating characteristic (ROC) curves estimated the sensitivity and specificity profiles of MMP-2 and MMP-9 for the identification of CRC.
Jeffery N., et al., 2009 [56]	MMP-1, 2, 3, 7, 9, 13, MT1-MMP, MT2-MMP and TIMP-1, TIMP-2, and IMP-3	Human adenomas and CRC	Immunohistochemistry	MMP-1, MMP-2, MMP-3, TIMP-1, and TIMP-2 showed a significant increase in carcinomatous epithelium compared with adenoma epithelium. The increased expression of MMPs and TIMPs occurred at an early stage of colorectal neoplasia.
Lièvre A., et al., 2006 [57]	The functional gene promoter polymorphisms of *MMP1*, *MMP3*, and *MMP7*	Human adenomas	Real-time PCR allelic discrimination assay	These data showed a relation between *MMP-1* -1607 ins/del G and *MMP-3* -1612 ins/del A combined polymorphisms and risk of small adenomas.
Tutton M.G., et al., 2003 [58]	MMP-2 and MMP-9	Patients’ plasma samples, adenomas, and CRC	Immunohistochemistry, real-time PCR, and ELISA	The expression of MMP-2 and MMP-9 was significantly increased in CRC tissues compared with matched normal tissues. Plasma MMP-2 and MMP-9 levels were significantly elevated at all stages in CRC patients. Plasma levels of these enzymes may be a noninvasive indicator of invasion or metastasis in CRC.

**Table 3:** MMPs are Regulated by microRNAs in CRC.

MicroRNA	MMP	Result
miR-34a	MMP-1, MMP-9	miR-34a overexpression prevents tumor cell proliferation, migration, and invasion [138,139].
miR-139	MMP-2	Downregulation of miR-139 reduces proliferation, migration, and invasion [140].
miR-29a	MMP-2	Upregulation of miR-29a increases metastasis [141].
miR-29b	MMP-2	Upregulation of miR-29b increases metastasis [142].
miR-143	MMP-7	Upregulation of miR-143 enhances tumor cell proliferation and invasion [143].
